# Serological report of pandemic (H1N1) 2009 infection among cats in Northeastern China in 2012-02 and 2013-03

**DOI:** 10.1186/1743-422X-11-49

**Published:** 2014-03-14

**Authors:** Fu-Rong Zhao, Chun-Guo Liu, Xin Yin, Dong-Hui Zhou, Ping Wei, Hui-Yun Chang

**Affiliations:** 1 State Key Laboratory of Veterinary Etiological Biology, Lanzhou Veterinary Research Institute, Chinese Academy of Agricultural Sciences, Lanzhou, Gansu Province 730046, People’s Republic of China; 2 State Key Lab of Veterinary Biotechnology, Harbin Veterinary Research Institute of Chinese Academy of Agricultural Sciences, Harbin, Heilongjiang Province 150001, People’s Republic of China; 3 Northeast Agricultural University, College of Veterinary Medicine, Harbin, Heilongjiang Province 150030, People’s Republic of China

**Keywords:** Pandemic (H1N1) 2009, Cats, Serological

## Abstract

****Background**:**

Influenza A virus has a wide range of hosts. It has not only infected human, but also been reported interspecies transmission from humans to other animals, such as pigs, poultry, dogs and cats. However, prevalence of A (H1N1) pdm09 influenza virus infections in cats in northeastern China is unknown. Therefore, the prevalence of A (H1N1) pdm09 influenza virus infections was performed among cats in northeastern China in this study.

****Findings**:**

Of all samples in this study, the overall seroprevalence of pandemic (H1N1) 2009 infection in cats was 21% (240/1140)**.** It also showed a higher prevalence rate of pandemic(H1N1) 2009 infection in pet cats (30.6%) than roaming cats (11%) based on NT. In addition, the results also showed a trend of difference in term of species of cats and it was statistically significant.

****Conclusions**:**

This is the first survey on the seroprevalence of pandemic (H1N1) 2009 infection among cats in northeastern China. This study has observed a relatively high seroprevalence of pandemic (H1N1) 2009 among different cat populations in northeastern China, similar seroprevalence studies should be conducted elsewhere.

## 

Influenza A virus has a wide range of hosts. Often the susceptibility of the species is dependent upon the characteristics of the virus and host. Numerous subtypes of influenza A viruses, including influenza A pandemic H1N1 2009 virus, have been shown to cross-species transmission. Since 2009, a novel influenza A virus (H1N1), now called A (H1N1) pdm09 influenza virus, has caused human influenza outbreaks in North America [1] and a worldwide pandemic [2-4]. To date, it has not only infected human, but also been reported interspecies transmission from humans to other animals, such as pigs, poultry, dogs [5-7].

Recently, the reports have shown that cats can also infected A (H1N1) pdm09 influenza virus [5,8]. Due to frequent cohabitation and close contacts with humans and other animals, cats are uniquely positioned to serve as reservoirs for influenza virus infection both within a household and within the larger farm or rural environment in China [9,10]. However, prevalence of A (H1N1) pdm09 influenza virus infection in cats in northeastern China is unknown. Therefore, the prevalence of A (H1N1) pdm09 influenza virus infections was performed among cats in northeastern China in this study.

A total of 1140 feline blood samples were collected from 56 different pet hospitals and four small animal shelters around northeastern China, from February 2012 to March 2013. The geographical and prevalent distribution of the samples has been concerned. Haerbin, Changchun and Shenyang were selected since they are the most densely populated area of commerce in northeastern China. Dalian was also included as it is the trade zone with large-scale breeding of poultry and pigs in northeastern China. The geographical location of serum samples of collection in northeastern China was displayed, please see the Figure 1. 660 blood samples from pet cats in hospitals and 480 blood samples from roaming cats were obtained. In each city, we selected the single largest small shelter. These serum samples were septed by centrifugation at 3,000 rpm for 15 min, and supernatants were transferred to a new eppendorf tubes and stored at-20°C until tested for antibodies against influenza A virus [11]. Additionally, in order to have a timely data for pandemic (H1N1) 2009 prevalence in northeastern China, 115 blood samples were retrospectively analyzed from pet dogs and pet cats in Harbin in 2008. All samples were tested by hemagglutination inhibition (HI) and Neutralization (NT) assay, according to the recommended procedures as previously reported [10,12]. HI titer ≥ 40 and NT titer ≥ 40 are considered as positive and indicate previous infection [12]. Influenza virus used in this study was A/California/7/2009(H1N1pdm09) [pandemic (H1N1) 2009 virus]. We additionally studied the sera for HI antibodies against three other viruses: a human seasonal H1N1 influenza virus A/Brisbane/59/2007(H1N1) and A/canine/Guangdong/2/2011(H3N2), a recently circulating H3N2 canine influenza virus (CIV) in dogs in China. The comparison of categorical variables between cat samples was performed with chi-square test where appropriate. Statistical significance was defined as p < 0.05. The data was analyzed with SAS software, version 9.1 [11].

**Figure 1:**
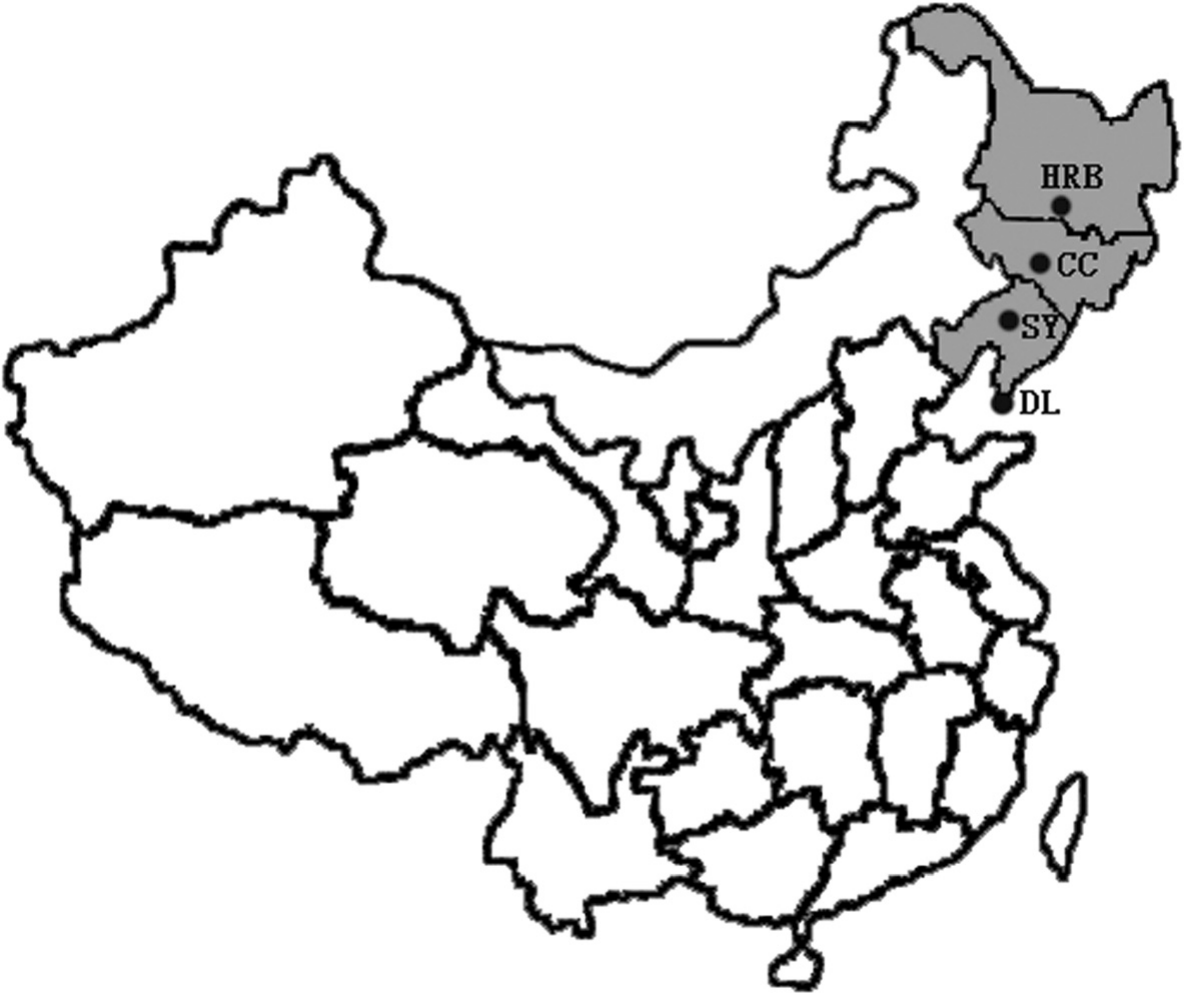
**Survey sites in northeastern China where the sero-study was conducted.** HRB: Haerbin; CC: Changchun; SY: Shengyang; DN: Dalian.

A total of 1255 serum samples were examined by NT and HI for pandemic (H1N1) 2009 antibodies. The serological screening revealed 21% pandemic (H1N1) 2009 infection in cats in northeastern China based on NT. It also showed a higher prevalence rate of pandemic (H1N1) 2009 infection in pet cats (30.6%) than roaming cats (11%) based on NT (p = 0.0032, Table 1). The results from HI also showed a trend of difference in term of species of cats and it was statistically significant (P = 0.002). The prevalence of the infection also showed a geographical difference in roaming cats as prevalent in Harbin and Changchun (20.8% and 23.3%) and absent in Shenyang and Dalian (Table 1). In addition, the factors of the gender and age of the cats were also analyzed as contributors to pandemic (H1N1) 2009 prevalence. In the Table 2, while no influence of age (seropositive data not shown) was found on cats infection with pandemic (H1N1) 2009, genders associated with the pandemic (H1N1) 2009 seropositivity by both HI and NT assay was significantly (p < 0.05). In addition, a total of 115 serum samples collected in 2008 had no HI or NT antibodies against A/California/7/2009 (data not shown). To rule out non-specific cross-reactivity, 1140 serum samples were titrated against seasonal influenza viruses (H1N1). Only twenty-four samples had a HI titer of 1:40 against H1N1 (Table 3). Only ten of these forty seasonal influenza positive-samples were also HI and NT positive for A/California/7/2009(H1N1pdm09). A total of 111 (9.7%) sera were positive by HI assay against H3N2 CIV (Table 3).

**Table 1:** Seroprevalence of pandemic (H1N1) 2009 in cats in different cities in northeastern China

**City**	**Roaming cats**				**Pet cats**			
	**No. examined**	**Seroprevalence (%)**		**OR(95%CI)**^**a**^	**No. examined**	**Seroprevalence (%)**		**OR(95% CI)**^**a**^
		**NT**	**HI**			**NT**	**HI**	
Haerbin	120	20.8%(25/120)	21.7%(26/120)	Reference	165	36.4%(60/165)	34.5%(57/165)	Reference
Changchun	120	23.3%(28/120)	20.8%(25/120)	1.05[0.57-1.95]	165	32.7%(54/165)	28.5%(47/165)	1.37[0.86-2.18]
Shenyang	120	0 (0/120)	0 (0/120)	67.58[4.06-1124]	165	27.9%(49/165)	30.3%(50/165)	1.21[0.76-1.93]
Dalian	120	0 (0/120)	0 (0/120)	67.58[4.06-1124]	165	23.6%(39/165)	21.2%(35/165)	1.96[1.20-3.20]
Total	480	11%(53/480)	10.7%(51/480)		660	30.6%(202/660)	28.0%(189/660)	
OR(95% CI)^b^		Reference	Reference			0.28[0.20-0.39]	0.30[0.21-0.41]	

^a^The statistical tests about the association between different cities in HI tests.

^b^The statistical tests about the association between roaming and pet cats;OR, odds ratio; CI, confidence intervals.

**Table 2:** Seroprevalence of pandemic (H1N1) 2009 in cats of different ages and genders, in northeastern China using the NT and HI assay

**Variable**	**Class**	**Positive rate**	
		**NT**	**HI**
Gender	Male	28.3%(163/576)	26.5%(153/576)
	Female	17.6%(99/564)	16.3%(92/564)
**Variable**^**A**^	**n**	**Unadjusted OR (95% CI)**	**Adjusted OR (95% CI)**
Raising pattern			
Age			
Less than 4 years	770	1.5 [0.71-1.93]	0.97 [0.66-1.39]
4 years and more	250	Reference	-
Gender			
Male	516	1.72 [1.12-1.99]	1.44 [0.91-1.70]
Female	504	Reference	-

^A^Unadjusted and adjust with logistic regression odds ratios for elevated HI and NT antibodies against pandemic (H1N1) influenza A virus among different cat populations in northeastern China.

**Table 3:** **Prevalence of elevated antibody titers against a canine influenza H3N2, a seasonal influenza H1N1, and A(H1N1)pdm09 among cats by hemagglutination inhibition (HI) assay, northeastern China**^**a**^

**HI assay virus**	**Number**	**Antibody titer**					**Number of specimens with titers ≥1:40 (%)**	
		**<1:20**	**1:20**	**1:40**	**1:80**	**≥1:160**		**OR(95% CI)**^**b**^
H3N2	1140	704	325	51	24	36	111 (9.7)	Reference
H1N1	1140	911	205	16	5	3	24(2.1)	5.02[3.20-7.86]
Pdm09	1140	518	382	123	52	65	240(21.5)	0.40[0.32-0.52]

^a^H1N1 = A/Brisbane/59/2007(H1N1); H3N2 = A/canine/Guangdong/2/2011(H3N2); Pdm09 = A/California/7/2009(H1N1pdm09).

^b^The statistical tests about the association between difference virus strains; OR, odds ratio; CI, confidence intervals.

Few seroprevalence studies on pandemic (H1N1) 2009 infections have been attempted in cats worldwide. The prevalence of this virus infection in cats in mainland China remains unknown. This is the first survey on the seroprevalence of pandemic (H1N1) 2009 infection in cats in northeastern China. Of all sera from cats in this study, 21% was identified as pandemic (H1N1) 2009 positive. In another conducting the seroprevalence of antibodies against (H1N1) pdm09 among cats in small cities of southern China was only 1.2% in 2011 [11]. Our increased antibody prevalence might be explained a number of ways. Perhaps cats were at a higher probability of infection in northeastern China, due to they exposures in dense populations of humans with high influenza A (H1N1) pdm09 attack rates. The difference might also be explained by the one year temporal difference between cats sampled in southern China in that the northeastern China cats had 1 more years to acquire influenza A (H1N1) pdm09 virus infection. Additionally, the prevalence of seropositive pandemic (H1N1) 2009 in male cats versus female cats suggests that the male cats may be more susceptible (P < 0.05) to the pandemic (H1N1) 2009 infections (Table 2). We hypothesize that relatively high A (H1N1) pdm09 transmission may have occurred between humans and cats during the period of virus infection in the human population. This hypothesis is supported by our observation that pet cats were more likely to have evidence of previous infection with A (H1N1) pdm09 that were roaming cats (30.6% vs11%, P = 0.0032) and also suggests a likely transmission between infected owners and their pets by close contact. Serological evidence of A (H1N1) pdm09 in domestic cats has been reported in the past. In a sero-survey conducted in Italy in 2009, a contrary low prevalence had been observed among dogs, while no cats were reported to have antibodies against A(H1N1)pdm09 in the screen [8]. A similar high prevalence of 21.8% and 22.5% were recorded in a population of cats in the United States, but the study sample comprised animals with a history of respiratory disease [10]. We hypothesized the sustained transmission of the influenza A (H1N1) pdm09 virus in the human population in our study area. In addition, it should be noted that 240 samples from the two small animal shelters in Harbin and Changchun had exposure to pandemic (H1N1) 2009 before sample collection. The higher prevalence of seropositive pandemic A (H1N1) pmd09 among Harbin and Changchun cats versus Shenyang and Dalian is unexplained.

Since cats may be exposed to different influenza virus subtypes, including human-avian and avian-origin influenza viruses, their potential role in the epidemiology of influenza virus should be further investigated. In summary, this study has observed a relatively high seroprevalence of pandemic (H1N1) 2009 in cats in northeastern China, similar seroprevalence studies should be conducted elsewhere. The studies showed that the prevalence for A (H1N1) pdm09 in human was correlated with age and population density. Preexisting antibody may have protected the very old from A (H1N1) pdm09 infection, while original antigenic sin and immunosenescence may have contributed to greater severity once infected [13-15]. Compare with all serum samples collected in 2008 had no HI and NT antibodies against A/California/7/2009, these results reflect the pandemic (H1N1) 2009 had been spread in cats. Concerns of rapid spread in small animal shelters and household may be needed. These observations highlight the need for monitoring cats in pet hospitals and small animal shelters are necessary for us to understand what roles cats plan in the ecology of influenza A virus.

## Competing interests

The authors declare that they have no competing interests.

## Authors’ contributions

FRZ and HYC designed the experiments. XY, DHZ carried out the test. PW,CGL and FRZ drafted the manuscript. All authors have read and approved the final manuscript.
